# Maternal pheochromocytoma and childbirth in Sweden 1973–2015: a population-based study on short and long-term outcome

**DOI:** 10.1007/s12020-024-03749-9

**Published:** 2024-02-29

**Authors:** Lisa Gunnesson, Oskar Ragnarsson, Maria Nilsson, Verena Sengpiel, Anders Elfvin, Erik Elias, Andreas Muth

**Affiliations:** 1 Department of Surgery, Region Västra Götaland, Sahlgrenska University Hospital, Gothenburg, Sweden; 2 Department of Surgery, Institute of Clinical Sciences, Sahlgrenska Academy, University of Gothenburg, Gothenburg, Sweden; 3 Department of Endocrinology, Region Västra Götaland, Sahlgrenska University Hospital, Gothenburg, Sweden; 4 Wallenberg Centre for Molecular and Translational Medicine, Gothenburg, Sweden; 5 Department of Obstetrics and Gynecology, Region Västra Götaland, Sahlgrenska University Hospital, Gothenburg, Sweden; 6 Department of Obstetrics and Gynecology, Institute of Clinical Sciences, Sahlgrenska Academy, University of Gothenburg, Gothenburg, Sweden; 7 The Queen Silvia Children’s hospital, Department of Pediatrics, Gothenburg, Sweden; 8 Department of Pediatrics, Institute of Clinical Sciences, Sahlgrenska Academy, University of Gothenburg, Gothenburg, Sweden

## Abstract

**Purpose:**

Data guiding management of pheochromocytoma and paraganglioma (PPGL) in pregnant women is limited, and long-term effects on the child are unknown. The aim of this retrospective registry-based case-cohort study was to assess how maternal PPGL and treatment impacts maternal and fetal outcome, including long-term outcome for the child. The main outcomes were maternal and fetal mortality and morbidity at delivery and relative healthcare consumption in children born by mothers with PPGL during pregnancy.

**Methods:**

The National Birth Register identified 4,390,869 pregnancies between 1973–2015. Data was crosslinked with three Swedish national registers to identify women diagnosed with pheochromocytoma or paraganglioma within one year before or after childbirth. Hospital records were reviewed and register data was collected for five age-matched controls for each child until age 18.

**Results:**

21 women and 23 children were identified (incidence 4.8/1.000.000 births/year), all women with adrenal pheochromocytomas (Pc). The majority (71%) were diagnosed post-partum. Nine women (43%) were hypertensive during pregnancy. Preterm delivery was more common in Pc patients compared to controls (30% vs 6%, *p* < 0.001). There was no maternal or fetal mortality. Timing of tumor removal did not affect gestational weight or APGAR scores. There was no observed difference in hospital admissions between children affected by maternal Pc and controls.

**Conclusion:**

Pc was commonly diagnosed after delivery and raised the risk of pre-term delivery, suggesting a need for an increased awareness of this diagnosis. However, reassuringly, there was no fetal or maternal mortality or any observed long-term impact on the children.

## Introduction

Pheochromocytomas (Pc) are rare, mainly non-metastatic tumors of the adrenal medulla, with an estimated annual incidence of 2–6/1,000,000 [1–3]. Sympathetic paraganglioma are extra-adrenal lesions that may present with similar symptomatology if hormonally active. The abbreviation PPGL is commonly used to describe the tumors as one clinical entity. The reported prevalence of PPGL among pregnant women is 1–2 cases per 100,000 pregnancies, but it is still an important cause of gestational hypertension [4–6]. As many as 5% of pregnancies are complicated by hypertension, most often attributed to gestational hypertensive disorders, e.g. pre-eclampsia, making PPGL easy to miss [7–10]. However, an undiagnosed PPGL may imply a considerable risk for mother and child with a historical maternal and fetal mortality rate of up to 50% [11], as uterine growth and delivery can cause uncontrolled catecholamine release from the tumor [5, 12]. More recent reports indicate decreasing mortality but still as high as 8 and 17% for mother and child respectively [6, 13, 14]. Most experts advocate minimally invasive surgery [15, 16], but whether it is preferable to remove a PPGL during early or late pregnancy has not been widely studied. Although it is generally recommended for mothers with PPGL to be delivered by caesarean section [12], recent reports indicate vaginal deliveries to be just as safe [13]. Whether there are any long-term health effects on the child is yet to be studied.

The primary aim of this national population-based study was to determine whether different treatment strategies for PPGL in pregnant women have impact on the outcome for mother and child in the peri-partum period. A secondary aim was to study hospital admissions during childhood and adolescence in children born after pregnancies complicated by maternal PPGL.

## Materials and methods

### Register search

The following mandatory national health registers were used to identify women diagnosed with either pheochromocytoma or paraganglioma, based on ICD (versions 7, 8, 9, and 10) and SNOMED coding (see Supplementary Table 1), within one year before or after childbirth:

- The Swedish Cancer Register - established in 1958 for national coverage of all tumor diagnoses. The register is considered to be of a high quality with an estimated coverage of 98–99% [17].
- The National Birth Register - covering all pregnancies leading to childbirth in Sweden since 1973, with near 100% coverage of all pregnancies from gestational week 28 + 0 up to June 30th 2008 and thereafter from gestational week 22 + 0 [18].
- The National Patient Register - established in 1964 for registration of all in-patient care, with complete national coverage since 1987 [19].
- The National Register of Causes of Death – registering causes of death according to international coding since 1951 [20].

We also crosschecked for any registered adrenalectomies during the same period to be sure not to miss any Pc registered under other diagnosis.

All register searches were made by the Swedish National Board of Health and Welfare based on personal identification numbers, unique for all Swedish citizens. Data for the mothers were delivered linked to personal identification numbers allowing for data validation. The children were linked to mothers and corresponding controls by code numbers and all analyses regarding the long-term follow up of the children were entirely anonymous and based on register data.

### Hospital charts

Further collection of hospital charts concerning the mothers was based on personal identification numbers, replaced by code numbers for all statistical analyses. Hospital records from surgical clinics performing adrenalectomies and/or other treatment for the patient’s tumor, as well as obstetric charts from the deliveries of interest were reviewed for information on timing of diagnosis, treatment of hypertension, and other PPGL related symptoms during pregnancy. Information on method and timing of delivery, surgical approach, and timing of the procedure was also collected. Outcome for both mother and child in terms of mortality, APGAR score [21], gestational weight and length, as well as any need of neonatal intensive care or other extra-ordinary interventions was studied. Any information concerning hereditary factors of interest, e.g. Multiple Endocrine Neoplasia (MEN) or Neurofibromatosis, was also collected, as were data on smoking, alcohol, drug abuse, previous pregnancies, and BMI.

### Child follow-up

Information on all diseases and hospital admissions registered for each child up to 18 years of age was collected from the National Patient Register and Cancer Register, regardless of ICD-coding. The same information was collected for five controls per child, chosen based on date of birth and county, with a random selection of five controls born at the same hospital within three months before or after the birth of each case. We chose to include only diagnostic codes provided in connection with hospital admissions as outpatient health-care visits were not included in the National Patient Register until 2001 [19].

### Ethics

The study was approved by the Ethical Review Board of Gothenburg on 05 May 2018 (Dnr 507-13). Informed consent was not considered necessary given the register-based design of the study.

### Statistics

SPSS version 24 was used for statistical analysis. Group comparisons were conducted by using non-parametric Mann–Whitney U test for independent samples. Correlation was calculated based on the Pearson correlation coefficient. *P*-values < 0.05 were considered statistically significant.

## Results

### Identification of patients

**Fig. 1:**
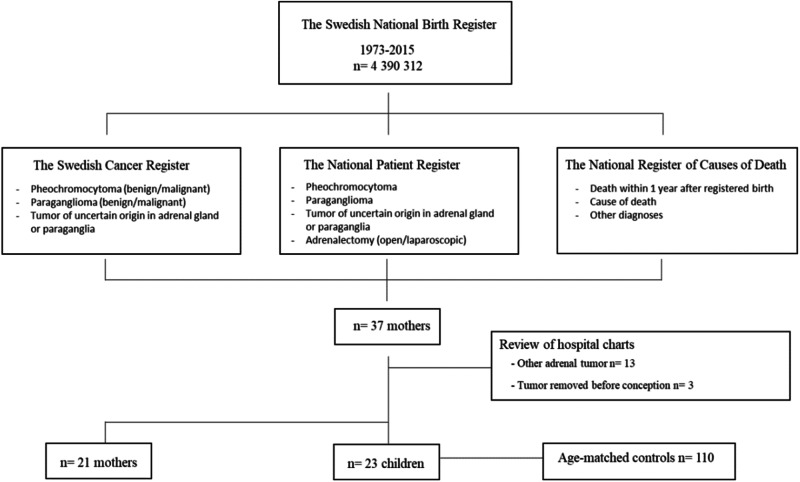
Cohort identification. The Swedish National Birth Register was crosslinked with three other national registers to identify women diagnosed with either pheochromocytoma or paraganglioma within one year before or after childbirth. Age-matched controls: Five controls per child born at the same hospital within three months before or after the birth were randomly selected from the Swedish National Birth Register

Out of 4,390,869 births registered in Sweden during 1973–2015, a total of 62 mothers, were identified in the Patient Register and/or the Cancer Register, either diagnosed with adrenal lesions or having undergone adrenalectomies in the specified period around delivery. No extra-adrenal paragangliomas were identified and the abbreviation Pc will therefore mainly be used. Thirty-eight adrenalectomies were performed for other diagnoses than Pc; adrenocortical adenoma (*n* = 23), adrenal cyst (*n* = 5), renal cancer (*n* = 3), metastasis (*n* = 2), hematoma (*n* = 1), adrenocortical carcinoma (*n* = 1), sarcoma (*n* = 1), ganglioneuroma (*n* = 1), other benign tumor (*n* = 1), and were excluded. Twenty-five of them after the initial register search, and the remaining thirteen after evaluation of hospital charts. Three patients were treated for Pc more than nine months before giving birth and therefore also excluded. Thus, 21 women were included in the analysis (Fig. 1). One mother delivered twins and one had given birth to another healthy baby only 11 months prior to the described delivery, finally resulting in 23 children of interest for further investigation. This corresponds to an annual incidence of 4.8/1.000.000 births [95% CI 3.1–6.7]. The Swedish population increased from 8,144,428 in 1973 to 9,851,017 in 2015, women of fertile age (15–44 years) increasing from 1,592,887 to 1,823,178. Total births per year were stabile over time, ranging from 84,422 to 122,014. When dividing the total observation time into four periods (1973–1983, 1984–1994, 1995–2005 and 2006–2015) the incidence of Pc during pregnancy, however, increased from 2.8 to 4.2, 4.9 and 7.3/1.000.000 births per year for each period respectively.

### Background data

The median maternal age at delivery was 28 years (range 18–37). Tumor size ranged from 15 to 150 mm (unknown in five patients) and catecholamine levels ranged from normal (*n* = 1) to >90 times the upper limit of normal. Ten tumors produced mainly epinephrine and three norepinephrine. In eight patients we could not find any data on hormone levels. Six patients had confirmed hereditary conditions predisposing Pc (29%). Five patients had MEN2 syndrome and one patient had a non-disease associated mutation of the RET gene. One patient had neurofibromatosis. Twelve patients presented with an apparently sporadic disease. In two cases it remains unknown whether they were sporadic or hereditary. Nine patients (43%) suffered from hypertension during pregnancy. In the entire cohort only six patients (29%) were diagnosed with Pc during pregnancy and they were all treated with alpha blockers. Three of these patients had hereditary disease (MEN2) and three were apparently sporadic (Table 1). The remaining three patients with hypertension, including the patient with known neurofibromatosis, were all presumed to have pre-eclampsia.

**Table 1:** Patient demographics

	Median (range)		*N* (%)
Age	28 (18–37)		
Hereditary		Total	7 (33%)
		MEN2	5 (24%)
		NF	1 (5%)
		Other	1 (5%)
		Unknown	2 (10%)
Tumor size (mm)	40 (15–150)		
Hormone profile		Epinephrine	10 (48%)
		Norepinephrine	3 (14%)
		Unknown	8 (38%)
Hormone xULN	3.6 (1–96)		
Hypertension		Yes	9 (43%)
		No	7 (33%)
		Unknown	5 (24%)
Alpha blocker		Yes	6 (29%)
		No	15 (71%)
Detection		During pregnancy	6 (29%)
		Post-partum	15 (71%)
Timing of surgery		Early pregnancy	1 (5%)
		Peripartum	4 (19%)
		Post-partum	16 (76%)

*MEN* multiple endocrine neoplasia, *NF* neurofibromatosis, *ULN* upper limit of normal range

Demographics of women diagnosed with pheochromocytoma affecting pregnancy in Sweden during 1973 – 2015

### Surgical treatment

Six patients (29%) were diagnosed during pregnancy. One of these patients was subject to tumor resection prior to delivery, later giving birth at full term. Four patients (19%) were scheduled for planned caesarian section in combination with open tumor resection in late pregnancy (gestational week 31–38). Sixteen patients (76%) had their tumors removed post-partum, only one of them diagnosed before delivery.

### Peri-partum outcome

Twelve (57%) deliveries were vaginal and 10 (43%) caesarean sections. Two of the caesarean sections were acute. There was no association between method of delivery and neonatal outcome.

Median gestational age at delivery was 38 + 0 weeks (range 31–42), with seven children (30%), including the two twins, being born before gestational week 37 + 0. Only three infants were born before week 34 + 0 (13%). Gestational hypertension was seen in all Pc affected mothers who delivered preterm and with the exception of the one duplex delivery being vaginal, all preterm births were through caesarean section, mainly due to maternal hypertension. No mother experienced hypertensive crisis or any other severe circulatory conditions in the peri-partum period.

Median birthweight was 3190 g (range 1770–4690 g) and only one child (4.3%) was born small for gestational age, compared to three in the control group (2.7%). There was no maternal or fetal mortality and APGAR scores were generally high with a median value of 9 at one minute and 10 at five minutes. However, there was a positive correlation between hypertension and decreased APGAR score after one minute, regardless of gestational age at birth (*p* 0.009). Two children had APGAR < 7 after one minute, both born during the 31st week of pregnancy due to maternal hypertension, however with normal weight for gestational age.

Interestingly, Pc affected children were not significantly smaller for gestational age, but preterm delivery (<37 weeks of gestation) was seen in 30% as opposed to 5.5% in controls (*p* < 0.001) and the reported frequency of respiratory distress (IRDS) was 13%, versus 1.8% in controls (*p* 0.010), see Tables 2 and 3. However, timing of tumor detection or removal did not seem to have any effect on gestational age, weight or APGAR scores, nor did tumor size or hormonal activity.

**Table 2:** General neonatal data

	Case (*n* = 23)	Control (*n* = 104)	*p*-value
Gestational age at birth (weeks)	38 (31–42)	40 (34–43)	*0.848*
Gestational weight (g)	3190 (1770–4690)	3550 (1900–5330)	*0.209*
Gestational height (cm)	48 (42–55)	51 (44–56)	*0.110*
APGAR 1	9 (5–10)	9 (2–10)	*0.851*
APGAR 5	10 (2–10)	10 (5–10)	*–*

General neonatal data on children and controls presented as median value and range

**Table 3:** Registered diagnoses up to 18 years

	Case (*n* = 23)	Control (*n* = 104)	*p*-value
Prematurity	7 (30%)	6 (5.5%)	<0.001^a^
SGA	1 (4.3%)	3 (2.7%)	0.680
IRDS	3 (13%)	2 (1.8%)	0.010^a^
Infections	4 (17.%)	16 (15%)	0.729
Psychiatric disorders	1 (4.3%)	3 (2.7%)	0.680
Total hospital encounters	1 (0–8)	0 (0–30)	0.087

Frequency of most common condition/diseases registered for each child and control up to the age of 18 years. Total number of hospital encounters presented as median and range. Significant *p*-values marked with^a^

*SGA* small for gestational age (≤−2SD), *IRDS* infant respiratory distress syndrome

### Long-term child outcome

Children to mothers with Pc were admitted for hospital care in median once (range 0–8), compared to nil for controls (range 0–30) during a median follow-up time of 12 years (range 1–19; *p* 0.09). The main reasons for hospital admissions in both groups were infectious diseases. Two children from mothers diagnosed with MEN2 were confirmed to have inherited the same genetic disorder and had been treated for medullary thyroid carcinomas. One of these was also diagnosed with a Pc as a teenager. No other cancers were found during follow up. The only cardiovascular diagnosis registered was arrythmia in a child in the control group born with pulmonary hypoplasia. For more detailed information, see Table 3.

## Discussion

In this Swedish population-based register study, we describe the management of pheochromocytoma in pregnant women in Sweden spanning four decades. Our main findings suggest a slightly lower incidence than previously reported and an underdiagnosis of Pc during pregnancy as 71% of the patients were diagnosed post-partum. However, the outcome was still favorable for both mothers and children. Preterm delivery was more common than in controls, but the Pc affected children were still not smaller for gestational age, nor did they consume more health care during childhood and early adolescence.

Our initial search was designed mainly to answer the question of the impact of choice of treatment strategy on the outcome for mother and child, rather than covering the total prevalence. The reported incidence of maternal Pc in the present study of 4.8/one million births per year is lower than those previously described [6, 13]. Biggar et al. performed a systematic review of articles describing Pc during pregnancy, with a risk of both publication and reporting bias. They chose to exclude all patients diagnosed later than one month post-partum, which may alter both incidence and mortality rates at least for the child. Bancos et al. combined a systematic review of case reports with an international, retrospective, multicenter study based on the International Pheochromocytoma and Pregnancy Registry and, therefore, potentially had a higher coverage. However, they excluded all case reports with fewer than five cases which may have altered the total incidence. Also, the coverage in the International Pheochromocytoma and Pregnancy Register varies between different regions. Swedish health care registers have almost 100% coverage and our reported incidence may therefore be considered population based. We have also, as opposed to previous authors [6, 13], deliberately chosen not to include previous pregnancies of the described women if registered more than one year prior to the pregnancy included in this study. We cannot assume with certainty that their tumors were already present and significantly hormonally active, even if this might have been the case. It is however also possible that previous studies have overreported some patients based on the assumption that a woman diagnosed with Pc during her third or fourth pregnancy had a hormone secreting tumor affecting her previous pregnancies as well. Our observed incidence rate increased with a factor 2.6 during the total study time which may reflect improved detection. Interestingly, and in contrast to previous studies, we found no patients with extra-adrenal paragangliomas. A possible explanation is the small cohort identified and the comparative rarity of extra-adrenal paraganglioma to Pc, but mis-coding and missed diagnoses cannot be excluded.

In the current study, most patients had their tumor removed post-partum, mainly due to late diagnosis. Pc is, in fact, a rare cause of hypertension but should always be taken into consideration as a differential diagnosis in hypertensive pregnant women, especially in absence of proteinuria and/or onset before the 20th gestational week [22, 23]. A number of described cases suggest that it is safe to resect Pcs either during late first or early second trimester [24], or at full-term pregnancy, either with concurrent tumor removal and caesarean section or postponed surgery after delivery [12, 25]. The rarity of this disease makes a prospective, randomized trial impossible to conduct but repeated reviews of large cohorts have come to similar conclusions without finding any significant difference in mortality when comparing early and late tumor resection [6, 26], in line with the findings of the current study.

Guidelines recommend alpha blockers to be initiated once the diagnosis is determined [6, 12]. In the present study, only six patients (28.6%) out of the total cohort of 21 were diagnosed with Pc during pregnancy and all six were treated with alpha blockers. The outcome was favorable for the entire cohort and did not differ based on use of alpha blockers. Recent studies have suggested pre-operative alpha blockers in non-pregnant Pc patients to be of less importance than previously implied, largely thanks to modern surgical and anesthesiologic techniques [27, 28]. In parallel, contemporary maternal and perinatal care in developed countries is generally safe, and perhaps the recommendation of referral of such patients to specialist centers and careful monitoring is likely more important than the alpha blocker per se [29].

Long-term treatment with alpha blockers results in prolonged catecholamine blockage and studies have shown some accumulation of phenoxybenzamine in the fetus, with up to 1,6 times higher concentration in blood from the umbilical cord than that of the mother [30, 31]. This should at least theoretically constitute a risk of impact on the newborn’s circulation as well as delaying its respiratory development, seeing that catecholamines play an important part in the production of surfactant in the alveoli of the lungs [32]. Accordingly, one could assume that for favorable fetal outcome the tumor should be resected soon after diagnosis, but this has not been proven to be of utter importance [6]. Fortunately, only a handful of children in this study needed respiratory assistance post-partum and in fact none of them were subjects to intrauterine alpha-blockage. Our small material does not allow us to draw any strong conclusions in this matter but rather strengthens the notion that the outcome for mothers and children of today is generally favorable, regardless of the use of alpha-blockers.

Catecholamines per se do not cross over the placental barrier [33] and the tumor influence on the fetus is thus primarily related to alterations in placental circulation, resulting in risks of hemorrhage, placental ablation, and fetal hypoxia [5]. Other causes of gestational hypertension may provoke the same alterations [9, 22], which can explain the observed correlation between maternal hypertension and low APGAR scores. Deteriorations in placental circulation also increase the risk of newborns being small for gestational age, and this has in fact been described in previous reports on Pc during pregnancy [34]. We saw no such tendencies in this study.

Gestational hypertension and preeclampsia have been shown to increase the risk of cardiovascular disease in the offspring [35–37], and we hypothesized that a maternal Pc may also increase the risk of childhood infections and/or other health issues but found no such predisposition. Preeclampsia of the mother has also been associated with higher BMI and impaired cognitive function in the child [37, 38]. We could not investigate this further in the present dataset as only diagnoses from hospital admissions during the first 18 years were analyzed. It would of course be interesting to further investigate whether they suffer from cardiovascular disease and/or obesity in a larger extent than the general population.

This study has strengths as well as limitations. The near 100% coverage of Swedish health care registers is a major strength, and our reported incidence may therefore be considered population based. Still, the identified cohort is small and we cannot exclude the possibility that patients with unfavorable outcomes could have been missed. Specifically, early miscarriages and abortions may not have been reported to the National Birth Register. Hence, data from this study cannot be used to inform on management and outcome of Pc during early pregnancy. However, the focus of this study was treatment strategies and outcome for mother and child in the peri-partum period, which is more accurately covered by our search. Even though the search was designed to detect both patients with Pc and patients with paragangliomas during pregnancy only patients with Pc were detected. This may be due to classification errors but is more likely explained by underdiagnosis, which was evident in mothers with Pc, along with the rarity of extra-adrenal paragangliomas [39].

In conclusion our data indicate that the incidence of pheochromocytoma during pregnancy may be lower than previously reported. Even though most patients were diagnosed with Pc only after delivery, there was no maternal or fetal mortality and despite the higher frequency of preterm delivery there were no observed negative long-term effects for the children. The timing of detection can often be of guidance concerning when to resect the tumor, and we believe it is safe to say that the hazard of being pregnant while harboring a pheochromocytoma has decreased markedly throughout the years.

### Supplementary information

Supplementary table 1

## Data Availability

No datasets were generated or analyzed during the current study.
