# Supplementary material for: Maternal pheochromocytoma and childbirth in Sweden 1973–2015: a population-based study on short and long-term outcome
Source: Endocrine. 2024 Feb 29;84(2):720–6. doi: 10.1007/s12020-024-03749-9 (PMC11076314; doi:10.1007/s12020-024-03749-9)
Supplement: Supplementary file 1 — Supplementary table 1 [file 12020_2024_3749_MOESM1_ESM.docx]

***Supplementary table 1.***

|  | ICD 7 | ICD 7/PAD | ICD 8 | ICD 9 | ICD 10 | SNOMED |
| --- | --- | --- | --- | --- | --- | --- |
| Pheochromocytoma | 1957 | 441 | 2260  2552 | 2270 | D35.0 | 87000 |
| Paraganglioma | 1950 | 441 | 2268 | 2276 | D35.6 | 86801 |
| Malignant pheochromocytoma | 1950 | 446 | 1925  1929  1940  1948 | 1940 | C74.1 | 87003 |
| Malignant paraganglioma | 1957 | 446 | 1948 | 1946 | C75.5 | 86803 |
| Tumor of uncertain malignant potential in adrenal gland |  |  | 2391  2550 | 2372 | D44.1 |  |
| Tumor of uncertain malignant potential in paragangliae |  |  | 2268  2382  2383  2384  2385  2389  2391 | 2373  2397 | D44.7 |  |

*Codes searched for in the Cancer Register, Patient Register and Register of Causes of death.*
